# Medicine

**Published:** 1909-09

**Authors:** J. R. Charles


### Progress of the Medical Sciences.

### MEDICINE.

**The relation between calcium metabolism, tetany and the parathyroids.**—Several recent researches tend to support the view that there is an intimate relation between the calcium metabolism of the body and tetany, between insufficiency of the parathyroids and tetany, and also that in some way the parathyroids influence the calcium metabolism of the body.

MacCallum and Voegtlin,[1] as a result of many extensive experiments and observations on dogs, draw the following conclusions : The effect of the experimental removal of the parathyroid glands in producing tetany may be annulled by the introduction of parathyroid extract, even from animals of very different character. The active principle is associated with a nucleoproteid, and may be separated from the remaining inert albuminous substances. This extract is not an iodine-containing compound.

The withdrawal of calcium salts leaves the nerve cells in a state of great hyperexcitability, which can, however, be made to disappear by giving a solution of a calcium salt. The injection of a solution of a salt of calcium into the circulation of an animal suffering from tetany promptly removes the symptoms and restores the animal to a normal condition ; while, on the other hand, the injection of potassium or sodium salts intensifies the state In human therapeutics the administration of calcium salts should be of use in the treatment of tetany to tide over the time during which the parathyroids are regaining their normal function, when this has been disturbed. Metabolism in parathyroidectomised animals shows a marked reduction in the calcium contents of the tissues, especially of the blood and brain during tetany, an increased output of calcium in the urine and fæces, an increased excretion of nitrogen in the urine, an increased urinary excretion of ammonia, with increased ammonia ratio in the urine and an increased amount of ammonia in the blood. These changes point to some form of acid intoxication, the symptoms of which are not, however, ameliorated by the administration of alkaline sodium salts. There appears to be a very considerable difference between the influence exerted by the thyroid and parathyroids on metabolism. In myxœdema there is a lowered metabolism, lowered bodily temperature, diminished respiratory changes, and diminished nitrogen excretion ; while in tetany the reverse of these is the case. It is possible that the absence of parathyroid secretion allows the formation of substances which, uniting with the calcium of the blood and tissues, promote its secretion.

Musser and Goodman,[2] as a result of their metabolic studies in a case of post-operative tetany which followed removal of the thyroid, seem inclined to take a somewhat different view from that expressed above, and point out that Stoeltzner[3] concluded that tetany is caused by too much calcium in the body, and also that Leopold and Von Reuss[4] found more calcium in the bodies of rats after injuring the parathyroids than in normal animals, and that these authors suggest that the poison which is normally neutralised by the parathyroids is able to precipitate calcium, leading to the deprivation of the tissues of physiologically active calcium. In

[1] *J. Exper. Med.*, Jan., 1909. [2] *Univ. Penna. M. Bull.*, May, 1909.
[3] *Jahrb. f. Kinderheilk.*, lxiii, 661. [4] *Wien. klin. Wchnschr.*, 1908, xxi. 1243.

their case the authors were not able to find any relation between the amount of calcium retention and the severity of the symptoms. Although they admit that calcium helps in keeping the symptoms of tetany in abeyance, they consider that the too rapid or too pronounced withdrawal of calcium from the body is as yet not proved to be the cause of tetany. They also point out that although many conditions, such as starvation, tuberculosis, diabetes, excessive muscular work, etc., cause a calcium withdrawal from the body, yet these states are not associated with tetany. The ammonia excreted in the urine in this case never, however, fell below 5 per cent., and the ammonia percentage was raised whenever the tetanic symptoms became worse. Taking into consideration all the facts of their case, they conclude that their patient was suffering from acidosis, and they place the blame on the parathyroids, suggesting that an acidosis may be the underlying feature of post-operative tetany.

The study of the effect of calcium salts in a case of gastric tetany, and of the parathyroids after death, has recently been made by Kinnicutt.[1] The patient, who was suffering from dilatation of the stomach secondary to duodenal ulceration and stricture, was admitted with severe tetany, for which he was treated with lavage and subcutaneous injections of normal saline, without any appreciable diminution of the spasms of the extremities. Later, parathyroid extract was administered without demonstrable beneficial effect; the following day two grains of calcium lactate in 1200 cc. of normal saline were given intravenously. "The immediate effect was to quiet the great restlessness of the patient, and a few hours later there was a marked diminution in the degree of spasm in the upper extremities, and for the first time since admission in the lower extremities." This beneficial result with regard to the tetany was maintained till the following day, when a further four grains was again given intravenously. "The patient fell asleep during its administration, and for the first time since admission he became entirely relaxed." The calcium treatment was further continued, and the symptoms of tetany remained in abeyance. Four days later the calcium was omitted, and a nucleo-proteid preparation of parathyroid injected into the gluteal muscles on two occasions. The spasms, however, recurred, to subside again to further administration of calcium. The patient throughout being too ill to be submitted to surgical operation, and being unable to retain food either in the stomach or rectum, died three days later from inanition and heart failure, without any further recurrence of tetany. After death the parathyroids were very carefully examined, but no abnormality was found on histological examination.

[2]A single parathyroid gland, even though injured, is sufficient to keep a dog from tetany, though if this be excised the animal

[1] *Am. J. M. Sc.*, July, 1909. [2] *J. Med. Research*, July, 1909.

dies of acute tetany. Experimental observations have been carried out with a view to ascertaining the practicability of transplantation of parathyroids to save a patient from tetany or death, following the removal of these bodies in parathyroidectomy. Many transplantation experiments have been successful in dogs, cats and rats, and several interesting and probably important results have come to light. In some of Halsted's experiments,[1] when too sudden parathyroid deficiency was created, a commencing tetany was tided over by the administration of calcium salts. Eiselberg[2] has apparently transplanted with success a human parathyroid into a woman who suffered from somewhat severe tetany following total thyroid extirpation.

Experimentally, Kocher[3] has prevented the onset of tetany in dogs after complete parathyroidectomy by transplanting thyroid tissue into the bone marrow of the tibia. When the bone containing the transplanted thyroid was removed, the animal quickly died from acute tetany. Further, in this resected tissue no evidence of any parathyroid tissue could be detected.

Thompson, Leighton and Swarts[4], working along the same lines, have transplanted parathyroid and thyroid tissue into the tibia in four dogs, following this by complete thyro-parathyroidectomy. All these animals passed the time limit without developing symptoms of tetany. In a second series only parathyroids were transplanted into the tibia, the result being the same as before, *i.e.* absence of tetanic symptoms. A piece of the thyroid transplanted subcutaneously in the neck did not prevent the occurrence of tetany in a dog on whom thyro-parathyroidectomy was performed. Further experiments showed in one case that after the insertion of a glass ball into the tibia total thyro-parathyroidectomy was not followed by tetany, and in two further experiments after tetany had been produced by removal of the thyroids and parathyroids that operations on the tibia without any transplantation of glands was followed by a cessation of the tetanic symptoms. Very curiously, therefore, bone injury in itself appeared to check or prevent the acute manifestations of tetany.

J. R. CHARLES.

### SURGERY.

The question of when and how to operate in cases of **exophthalmic goitre** is still in this country, I believe, unsettled, and judged by the diverse views expressed by those in other countries the same condition holds in them, though they appear to operate much more freely than we do. Now that the disease is definitely known to be due to an excessive secretion by, and an excessive

[1] *J. Exper. Med.*, No. 1, 1909. [2] *Wien. klin. Wchnschr.*, No. 21, 1907.
[3] *Arch. f. klin. Chir.*, No. 1, 1908. [4] *J. Med. Research*, July, 1909.

